# Percutaneous CT-Guided Microwave Ablation Combined with Pedicle Screw Fixation Followed by Vertebroplasty (MASFVA): Initial Experience of a Minimally Invasive Treatment of Vertebral Metastases with Extension to the Vertebral Pedicle

**DOI:** 10.3390/curroncol30020127

**Published:** 2023-01-30

**Authors:** Claudio Pusceddu, Salvatore Marsico, Daniele Derudas, Nicola Ballicu, Luca Melis, Carlo de Felice, Alessandro Calabrese, Domiziana Santucci, Eliodoro Faiella

**Affiliations:** 1 Department of Oncological and Interventional Radiology, Businco Hospital, Via Edward Jenner 1, 09121 Cagliari, Italy; 2 Department of Radiology, Hospital del Mar, Pg. Marítim de la Barceloneta, 25, 29, 08003 Barcelona, Spain; 3 Department of Hematology, Businco Hospital, Via Edward Jenner 1, 09121 Cagliari, Italy; 4 Nuclear Medicine Department, Businco Hospital, Via Edward Jenner 1, 09121 Cagliari, Italy; 5 Department of Radiological Sciences, Oncology and Pathology, Umberto I Hospital, Sapienza University of Rome, Viale del Policlinico 105, 00161 Rome, Italy; 6 Department of Radiology, Sant’Anna Hospital, Via Ravona 20, San Fermo della Battaglia, 22042 Como, Italy; 7 Unit of Computer Systems and Bioinformatics, Department of Engineering, Campus Bio-medico University, Via Alvaro del Portillo 21, 00128 Rome, Italy

**Keywords:** spine metastases, microwave ablation, percutaneous therapies, screw fixation, interventional radiology, vertebroplasty

## Abstract

(1) Background: The aim of this study was to retrospectively evaluate the safety and efficacy of a combined CT-guided percutaneous microwave ablation (MWA) and pedicle screw fixation followed by vertebroplasty (MASFVA) for the treatment and stabilization of painful vertebral metastases with vertebral pedicle involvement. (2) Methods: from January 2013 to January 2017 11 patients with 16 vertebral metastatic lesions (7 men and 5 women; mean age, 65 ± 11 years) with vertebral metastases underwent CT-guided microwave ablation and screw fixation followed by vertebroplasty (MASFVA). Technical success, complication rate, pain evaluation using a visual analogue scale (VAS), Oswestry Disability Index (ODI) and local tumor control were examined. (3) Results: Technical success rate was 100%. No procedure-related major complications occurred. VAS score decreased from 6.8 ± 0.7 to 0.6 ± 0.6. ODI score decreased from 3.1 ± 0.7 to 1.2 ± 0.4. All patients could walk independently without neurological complication after one week from the procedure. No new bone fractures or local disease recurrence occurred during a median follow-up of 12 months. (4) Conclusions: Our results suggest that MWA and percutaneous pedicle screw fixation followed by vertebroplasty for the treatment of painful vertebral metastases is a safe and effective procedure for painful vertebral metastases with vertebral pedicle involvement, allowing pain relief and local tumor control.

## 1. Introduction

Vertebral metastatic disease is a very common and difficult “scenario” that causes severe morbidity in cancer patients, primarily due to the progressive onset of pain, instability, and neurologic deficit.

Vertebral osteolytic metastases, in particular, cause pain and pathological fractures, with a significant reduction in quality of life and mobility. Most bone metastases have a negative impact on the patient’s short-term prognosis because bone lesions are rarely completely eradicated.

Due to the poor response to conventional conservative therapies (chemotherapy, radiotherapy, hormone therapy, bisphosphonates, and analgesics) and the frequent contraindications to surgery, interventional radiology (IR) treatment can play a critical role [1,2]. Several IR modalities can be used alone or in combination for the local treatment of bone lesions, with varying goals ranging from palliative to tumor debulking and injured bone stabilization.

Vertebroplasty and kyphoplasty can help with bone lesion pain, but are usually not effective for mechanically unstable pathologic fractures involving the middle and posterior vertebral column. In these cases, it is necessary to perform a posterior screw fixation for structural spine stabilization.

Once the patient has been selected, a pre-procedure study will be performed, and the most appropriate technique will be determined based on several factors such as the tumor’s structural features, extension, localization, and relationships with adjacent tissues. Radiofrequency, alcohol, interstitial laser, microwave, and cryoablation are all common ablative procedures [3,4,5,6,7,8,9,10,11,12,13].

The purpose of the study is to evaluate the safety and efficacy of a novel combined technique of microwave ablation and CT-guided screw fixation followed by vertebroplasty (MASFVA) for the treatment of refractory painful vertebral metastases with an involved pedicle to enable synchronous local tumor debulking, pain reduction, and overall structural stabilization of the spine.

## 2. Materials and Methods

This is a retrospective observational study; only existing information collected from human participants was used, and there are no identifiers linking individuals to data/samples. Institutional Review Board approval was obtained. All methods and procedures conformed to the ethical standards of the institution and the research committee, in accordance with the 2013 Declaration of Helsinki. Informed consent was obtained from all individual participants included in the study.

Eleven patients (7 men and 4 women; mean age 65 ± 11 years) with 16 vertebral metastatic lesions (5 non-small-cell lung cancer, 3 breast carcinoma, 2 small-cell lung cancer, and 1 thymoma) who underwent CT-guided microwave ablation and screw fixation followed by vertebroplasty (MASFVA) between January 2013 and January 2017 were included in the study. After 2017, we did not identify patients eligible for the procedure. Follow up and data collection were skipped in the period from 2019 to 2022 due to the COVID-19 pandemic. All patients had previously undergone radiation therapy in combination with chemotherapy. The clinical data of the patients included in the study are shown in Table 1.

The inclusion criteria were as follows: (a) presence of a lesion with evidence of histologic malignancy of vertebral metastasis that resulted in mechanical instability (infiltration of one or two pedicles), was radioresistant, adjacent to irradiation-sensitive structures and/or did not respond to chemotherapy at least 3 weeks prior to the ablation session; (b) complications associated with chemotherapy that required treatment discontinuation; (c) life expectancy greater than 2 months; and/or (e) ineligibility for surgical treatment and intractable back pain unresponsive to continuous treatment with opioids and nonsteroidal anti-inflammatory drugs (NSAIDs).

The exclusion criteria were presence of primary spinal tumor, diffuse metastatic spinal disease, complete vertebral collapse (vertebra plana), extensive epidural and spinal canal infiltration (more than a third of the extension of the circumference of the epidural space), and moderate and severe neurologic deficits.

The preoperative evaluation consisted of a combined oncology–radiology interventional clinical examination in the outpatient setting of interventional radiology. Pain severity was measured using the visual analog scale (VAS), which continuously rates pain on a scale of 0 to 10 to indicate pain intensity, and using the Oswestry Disability Index (ODI), with a score ranging from 0 to 5, where 0 corresponds to no difficulty or pain, while 5 corresponds to inability to perform daily activities. Both VAS score and ODI were performed in the follow-up of patients at 1 and 3 months after surgery.

All patients underwent contrast-enhanced CT or MRI prior to the procedure to assess the location, size, and radiological characteristics of the lesions to proceed to intervention planning. The procedure was performed in a single vertebra in eight patients and in two vertebrae in three patients.

The vertebral approach was unilateral with a single screw in four patients and bilateral with two screws in the remaining seven.

A non-enhanced CT scan was performed immediately following the procedure to evaluate the results and any complications.

Contrast-enhanced CT scans were acquired 3, 6 and 12 months after the procedure to perform radiological follow-up.

If the patient showed persistence of pain, a complete X-ray of the spine in antero-posterior and lateral-lateral projection was also performed the day after the procedure to assess the overall biomechanical status of the spine.

Drug therapy (NSAIDs and opioids) was discontinued 1 week after treatment and resumed in case of persistence or exacerbation of pain symptoms.

### 2.1. Treatment Technique

Percutaneous bone microwave ablation (MWA) was performed under dual CT and fluoroscopic guidance to allow precise needle placement, increased operator comfort, proper visualization of fixation and local complications, and to avoid and monitor any leaks during vertebroplasty.

CT acquisition data were: 5 mm collimation at 80–140 mA (CT system: SOMATOM Sensation, Siemens, AG, Forchheim, Germany).

The patients, in the prone position, underwent conscious sedation with continuous intravenous infusion of fentanyl citrate (0.1 mg/2 mL diluted 1:10 with saline) and received local anesthesia including subcutaneous injection of 2% lidocaine hydrochloride. Anesthesia was planned in each case by the anesthesiologist who examined the patient prior to the procedure. Depending on the patient’s condition, the anesthesiologist could change the type of sedation (mild or deep). Pain was monitored according to vital signs.

Preoperative antibiotic (a single dose of cefazolin 2 g) was administered intravenously 20–30 min before treatment.

Percutaneous MWA was performed using a 2.45 GHz microwave generator (AMICA-GEN, HS Hospital Service, Aprilia, Italy) delivering energy through a 14-gauge, minichoked, water-cooled interstitial antenna (AMICA-GEN).

If the metastasis was confined to the pedicle or ipsilateral half of the vertebral body, the lesion was effectively ablated using a single 14-gauge antenna.

If the lesion exceeded the midline of the vertebral body, 2 MW antennas with bipedal access were placed to allow the best possible ablation of the entire lesion. The antenna length used for ablation was 20 cm in all cases.

To preserve heat-sensitive anatomical structures, such as the spinal cord or nerve roots, one or more thermocouples were placed in the adjacent epidural space before starting ablative treatment.

After insertion of the antenna into the lesion through a transpedicular access, the introducer was retracted to avoid interfering with microwave emissions from the active tip and to prevent overheating of the cannula during ablation, then energy delivery was initiated. During the ablation procedures, the thermocouple recorded no dangerous temperature rise (>45 °C) [14].

In 15% of the sessions, a single antenna was used (*n* = 3), while in 85% (*n* = 19) two antennas were used.

After ablation, the infiltrated pedicle was stabilized by percutaneous placement of a cannulated screw and subsequent vertebroplasty.

For the vertebral transpedicular approach, we used screws of 4.5 mm in diameter and 4–6 cm in length.

The pedicle was percutaneously cannulated with a bone biopsy needle.

After the vertebroplasty cannula was positioned correctly, a k-wire was inserted into the cannula, and then the vertebroplasty cannula was removed.

After a small skin incision, we inserted the screw using the Kirschner wire as a guide to place the screw in its final position.

Ablation treatment was always combined with vertebroplasty, achieved by injecting polymethyl methacrylate (PMMA) through the cannulated screw that has holes on both sides and at the tip (average volume, 3.5 mL; range 3–5 mL).

In patients who had very extensive destruction of the vertebral body or in cases of bilateral involvement of both pedicles, we performed treatment with the insertion of two screws in the same session.

If the lesion exceeded the midline of the vertebral body, a vertebroplasty was also performed in these lesions to allow adequate vertebral cementation.

In 4 cases, screws were placed in both infiltrated pedicles, while in the remaining 7 cases, only one screw was placed.

After the procedure, a non-enhanced CT scan was performed, and the patients were immediately transferred to the recovery room for observation.

### 2.2. Statistical Analysis

For the purpose of this study, continuous variables were reported as mean ± standard deviation (SD). Differences between mean VAS and ODI scores at baseline and at 1, 3, 6 and 12 months post-procedure were assessed by Student’s exact test or Fisher’s exact test, as appropriate. A *p* value less than 0.05 was considered significant. Statistical analysis was performed with OpenStat software.

## 3. Results

In all cases, we observed the technical success of the MASFVA technique, defined as the ability to complete the procedural sequence of vertebral lesion ablation with correct screw placement without significant cement leakages. Minimal cement leakage, defined as para-vertebral, small, and asymptomatic, occurred in 3 out of 11 patients (27%), with no clinical repercussions.

The overall mean duration of the percutaneous microwave ablation procedure followed by screw fixation and vertebroplasty was 63 ± 18 min.

**Figure 1:**
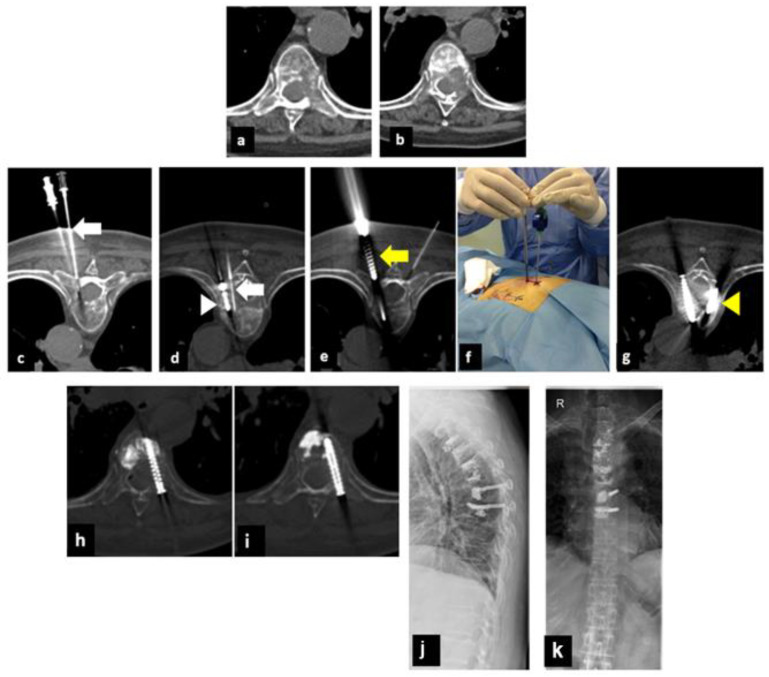
A 67-year-old woman with a history of non-small cell lung cancer, previously treated with multiple dorsal vertebroplasties for pathological vertebral fractures. (**a**,**b**): Axial CT shows extensive osteolytic metastases of the D7 and D8 vertebral bodies that involved the right pedicle in both cases. (**c**,**d**): Axial CT shows thermocouple placement (white arrows) and thermal ablation with MW (arrowhead). (**e**,**f**): Axial CT scan and intraoperative image (**f**) show the positioning of the screw (yellow arrow). (**g**): Axial CT scan shows contralateral placement of the vertebroplasty cannula. h-i-l-m: Axial CT scan shows post-procedure control (**h**,**i**) and a radiograph of the thoracolumbar spine at 3 months in antero-posterior and latero-lateral projection (**j**,**k**) after microwave ablation combined with pedicle screw fixation followed by vertebroplasty (MASFVA).

**Figure 2:**
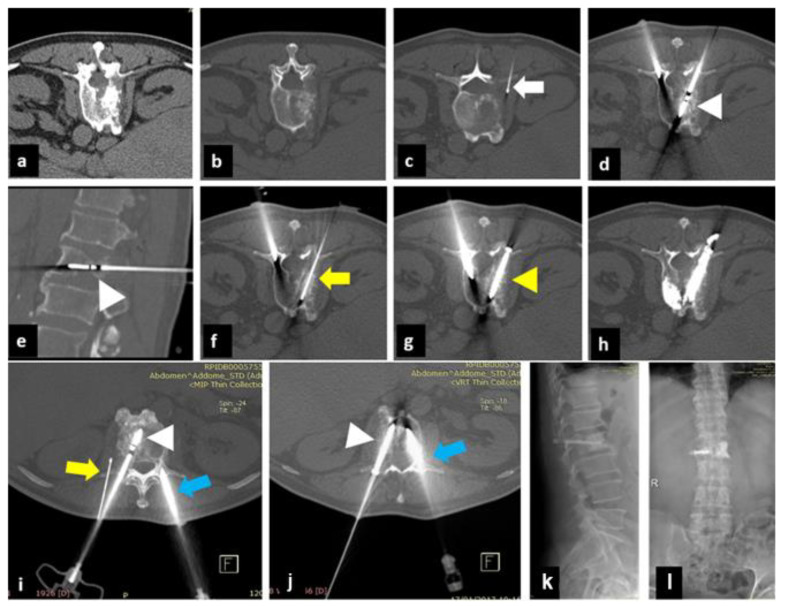
A 65-year-old man with a history of small cell lung carcinoma. (**a**,**b**): Axial CT shows extensive osteolytic metastasis of the L2 vertebral body involving the right pedicle. (**c**): Axial CT shows thermocouple placement in the left root between L2 and L3 (white arrow). (**d**,**e**): Axial and sagittal CT, MW antenna placement in the tumor for thermal ablation. Contralateral initial placement of the VTP needle (white arrow). (**f**): Axial CT shows k-wire placement for subsequent coaxial screw insertion (yellow arrow). (**g**): Axial CT shows placement of the left screw (yellow arrow) and contralateral advancement of the vertebroplasty cannula (blue arrow). (**h**): Axial CT shows cementing of the screw (yellow arrowhead) and contralateral vertebroplasty (blue arrow). (**i,j**): Axial CT with Maximum Intensity Projection (MIP) reconstruction showing the MW antenna (white arrow) and thermocouple (yellow arrow) positioned and the vertebroplasty cannula in contralateral extrapedicular location (blue arrow). (**k**,**l**): Postoperative 3-month radiograph of the thoracolumbar spine in antero-posterior and latero-lateral projection after microwave ablation of the L2 vertebra combined with pedicle screw fixation followed by vertebroplasty (MASFVA).

**Figure 3:**
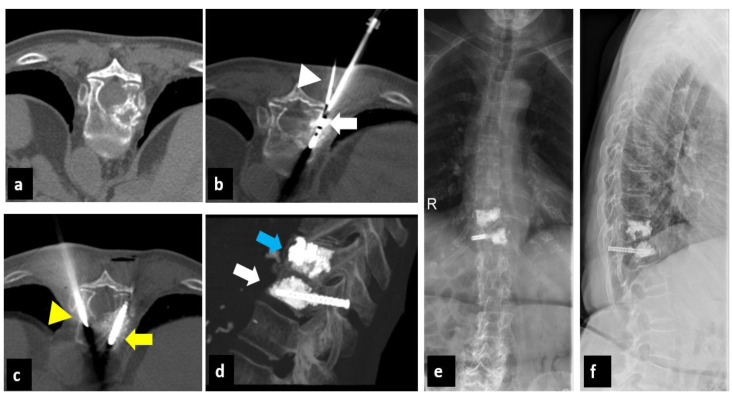
A 84-year-old woman with history of breast cancer, treated with simple vertebroplasty for pathologic D11 vertebral fracture 3 years earlier. (**a**): Axial CT scan shows osteolytic metastasis of vertebral body D12 involving the right pedicle. (**b**): Axial CT shows placement of the thermocouple (white arrowhead) in the right anterior epidural space and transpedicular placement of the MW antenna (white arrow) in the D12 vertebral body for thermal ablation. (**c**): Axial CT shows the placement of the right screw (yellow arrow) and contralateral advancement of the vertebroplasty cannula (yellow arrowhead). (**d**): Postoperative sagittal CT with Maximum Intensity Projection (MIP) reconstruction after microwave ablation of D12 vertebra combined with pedicle screw fixation followed by vertebroplasty (white arrow). Vertebroplasty of D11 performed 3 years earlier (blue arrow). (**e**,**f**): Radiograph of the thoracolumbar spine 3 months after surgery, in antero-posterior and latero-lateral projection, after microwave ablation of the D12 vertebra combined with pedicle screw fixation followed by vertebroplasty (MASFVA).

Post-procedure CT scans (without contrast enhancement) showed no major complications, such as bleeding, incorrect screw position, or cement leakage (Figure 1, Figure 2 and Figure 3).

Only 2 of the 11 patients (18%) complained of moderate pain on the postoperative day.

All patients were discharged 24 h after treatment in stable condition and without complications.

One week after the procedure, in clinical evaluation, all patients could walk independently and showed no signs of neurological complications. This condition persisted during the median follow up time of 1 year. Mean VAS pain assessment score on the day before treatment was 6.8 ± 0.7 (range, 6–8).

One month after treatment, the median VAS pain score was 1.7 ± 1.0 (range, 1–4) with a mean reduction of 75% (6.8 ± 0.7 vs. 1.7 ± 1.0; *p* < 0.000).

At the 3-month assessment, the median VAS pain score was 0.6 ± 0.6 (range, 0–2) with a mean reduction of 92% (6.8 ± 0.7 vs. 0.6 ± 0.6; *p* < 0.000) compared with the baseline assessment.

Mean ODI disability assessment score on the day before treatment was 3.1 ± 0.7 (range 2–4).

One month after treatment, the median ODI score of disability was 1.4 ± 0.4 (range, 1–2) with a mean reduction of 55% (3.1 ± 0.7 vs. 1.4 ± 0.4; *p* < 0.000).

**Figure 4:**
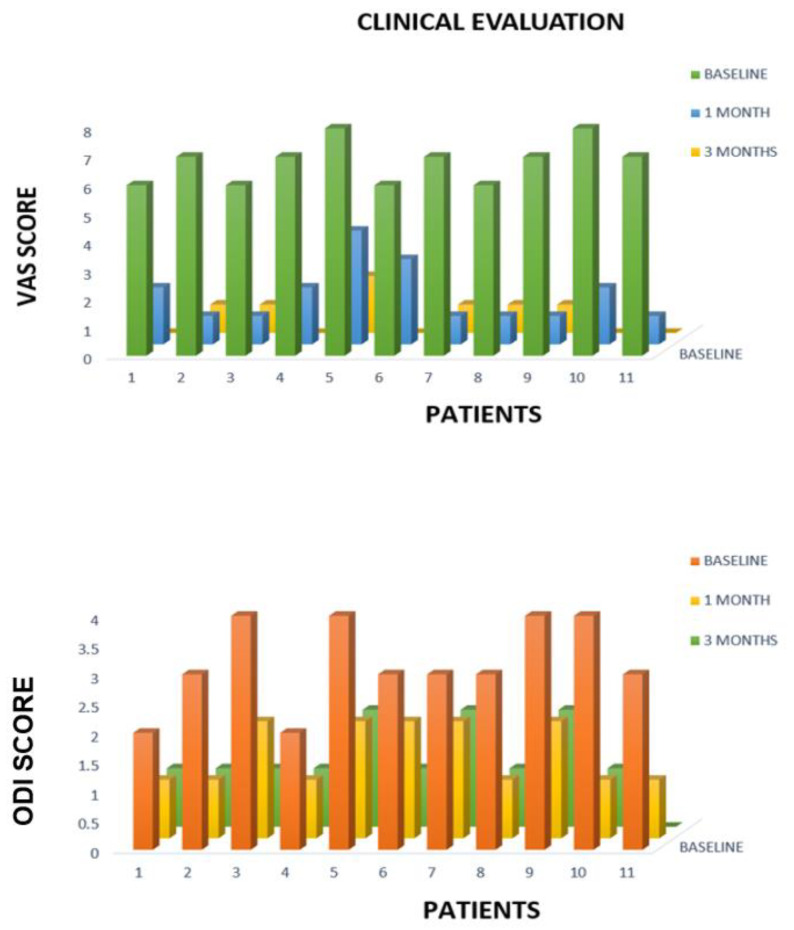
Comparison of VAS and ODI score at baseline, 1 month and 3 months after the procedure.

At the 3-month assessment, the median ODI score for disability was 1.2 ± 0.4 (range, 1–2) with a mean reduction of 62% (6.8 ± 0.7 vs. 0.6 ± 0.6; *p* < 0.000) compared with the baseline assessment (Figure 4).

No infectious complications were observed during follow-up.

None of the patients died from disease progression during the considered follow-up.

Contrast-enhanced CT scans performed 3, 6, and 12 months after the procedure showed no local recurrence, loosening of the screws, or new fractures in the treated site.

## 4. Discussion

The skeleton is the third most frequent site for metastatic localization, ranking third after the lung and liver, and metastatic bone disease is the most common malignant disease of the bone.

In addition, spinal metastases are the most frequently encountered spinal tumor, can affect up to 50% of cancer patients, and cause various symptoms and disability.

When metastases are localized to the spine, painful or pathological fractures may be accompanied by instability and severe clinical disability when the lesion extends to the posterior spine [15].

In the treatment of vertebral metastases there are many systemic clinical tools that are commonly used, and the main ones are chemotherapy, radiotherapy, hormone therapy, bisphosphonates, and analgesics [1].

Open surgical treatment, which consists of decompressive laminectomy with long-level screw fixation and bone fusion, is indicated in cases of obvious spinal instability, clinically significant neural compression secondary to retropulsed posterior somatic wall or spinal deformity, intractable pain unresponsive to nonsurgical measures, and radiotherapy failure, but it is often of limited benefit in the management of spinal metastases due to its morbidity [2].

Some histological forms of bone metastases, such as those arising from sarcoma, renal cell carcinoma, non-small cell lung carcinoma, and melanoma, respond less well to radiotherapy [16]. Furthermore, the use of radiotherapy is limited by spinal cord cumulative dose tolerance [17].

In a previous study, we have already evaluated as effective and safe the use of combined treatment of radiofrequency ablation with a steerable device and vertebroplasty for the management of painful bone metastases, resulting in improved patient quality of life [18].

A systematic review of the literature conducted by Sagoo et al. [19] on the use of MWA, whether or not associated with vertebroplasty, for the treatment of patients with painful vertebral metastases showed promising results, as several studies demonstrated the ability of MWA to achieve local tumor control, defined as no evidence of tumor progression at last follow-up [20,21,22]. At the end of 1-year follow-up, we achieved optimal results, as there were no new bone fractures or local disease recurrences detected by contrast-enhanced CT.

In our study, vertebroplasty has always been performed because of the intrinsic characteristics of cement both in reducing tumor, due to the cytotoxic effect from the direct exothermic reaction of cement, and in reducing pain because, together with screw fixation, it allows us to reduce pain by stabilizing fractures by acting on the interosseous sensitive fibers [23,24].

However, vertebroplasty usually has a limited role in mechanical support and is not effective in treating unstable spinal metastases that extend to the posterior elements, and posterior instrumentation must be inserted to stabilize the spine due to advanced cancer-related instability [25]. Giammalva et al. studied a combined approach with radiofrequency ablation and vertebroplasty followed by screw fixation, determining it a safe and effective technique for the treatment of metastases to the spine, but with a lower mean VAS pain score at 3 months after the procedure compared to our study (3.35 vs. 0.6) [26].

The novelty of our study concerns percutaneous screw fixation, as there are no studies in the literature regarding the combined approach of MWA and percutaneous pedicle screw fixation followed by vertebroplasty. Screw fixation is increasingly used as a minimally invasive method of fixation for the treatment of lumbar and thoracic spine disorders and is now performed percutaneously with interventional radiology techniques using cannulated screws [27]. Percutaneous stabilization with transpedicular screws, whether associated with vertebroplasty or not, has been performed because, as shown in several studies, in cases of extension of the lesion to the posterior spine, it improves pain relief, spinal stability, surgical stress, and postoperative survival time, and reduces the evidence of fractures secondary to vertebroplasty [28,29]. The screws have a central core that allows cement to pass through, with holes at the sides and the tip that ensure cement entry into the pedicle. This further increases the stability of the pedicle, reducing the risk of pathologic fractures.

Our study has some limitations. It is a retrospective study, and only patients who underwent the combined MWA and pedicle screw fixation followed by vertebroplasty were included, so a comparison between the outcomes of this novel procedure and other approaches was not feasible. The small sample size and the short follow-up period did not allow the evaluation of late oncologic outcomes and quality of life.

However, we believe that this is a promising new approach for the minimally invasive treatment of spine metastases combining tumor debulking, pain reduction, and increased stability of the spine.

## 5. Conclusions

This preliminary result suggests that combined MWA and pedicle screw fixation followed by vertebroplasty is a safe and effective procedure which allows us to reduce the tumoral tissue, stabilizing the vertebral metastasis with significant pain relief and good recovery of walking capacity. 

This technique seems to be a promising alternative for patients with pain and unresponsive to conventional treatments that are not candidates for surgery. 

However, further studies with large series are required to confirm these preliminary results.

## Figures and Tables

**Table 1:** Histology of primary malignancies, topographic distribution of tumor lesions, devices used and clinical evaluation of the study population.

Patient	Age (years)	Primary Tumor	Sites of Treatment	Number of Screws	Number of Microwave Probes	Baseline VAS Score /ODI Index	1 Month VAS Score /ODI Index	3 Months VAS Score /ODI Index
1	57	breast cancer	L1	1	1	6/2	2/1	0/1
2	40	thymoma	L1	1	2	7/3	1/1	1/1
3	70	NSCLC	D8–D9	2	2	6/4	1/2	1/1
4	67	NSCLC	D7–D8	2	2	7/2	2/1	0/1
5	74	SCLC	L1	1	1	8/4	4/2	2/2
6	50	NSCLC	D10	2	2	6/3	3/2	0/1
7	84	breast cancer	D11	1	2	7/3	1/2	1/2
8	72	NSCLC	D11	2	2	6/3	1/1	1/1
9	70	NSCLC	D12	1	2	7/4	1/2	1/2
10	64	breast cancer	L3	2	2	8/4	2/1	0/1
11	65	SCLC	L2	1	1	7/3	1/1	0/1

NSCLC nonsmall cell lung cancer, SCLC small cell lung cancer, VAS Visual Analog Score, ODI Oswestry Disability Index.

## Data Availability

The data presented in this study are available on request from the corresponding author.
